# Vascular tube formation on matrix metalloproteinase-1-damaged collagen

**DOI:** 10.1038/sj.bjc.6604357

**Published:** 2008-04-29

**Authors:** J Varani, P Perone, R L Warner, M K Dame, S Kang, G J Fisher, J J Voorhees

**Affiliations:** 1 Department of Pathology, University of Michigan Medical School, Ann Arbor, MI 48109, USA; 2 Department of Dermatology, University of Michigan Medical School, Ann Arbor, MI 48109, USA

**Keywords:** Tumor invasion, inflammation, wound healing, angiogenesis

## Abstract

Connective tissue damage and angiogenesis are both important features of tumour growth and invasion. Here, we show that endothelial cells maintained on a three-dimensional lattice of intact polymerised collagen formed a monolayer of cells with a cobblestone morphology. When the collagen was exposed to organ culture fluid from human basal cell tumours of the skin (containing a high level of active matrix metalloproteinase-1 (MMP-1)), degradation of the collagen matrix occurred. The major degradation products were the $3over 4$- and $1over 4$-sized fragments known to result from the action of MMP-1 on type I collagen. When endothelial cells were maintained on the partially degraded collagen, the cells organised into a network of vascular tubes. Pretreatment of the organ culture fluid with either tissue inhibitor of metalloproteinase-1 (TIMP-1) or neutralising antibody to MMP-1 prevented degradation of the collagen lattice and concomitantly inhibited endothelial cell organisation into the vascular network. Purified (activated) MMP-1 duplicated the effects of skin organ culture fluid, but other enzymes including MMP-9 (gelatinase B), elastase or trypsin failed to produce measurable fragments from intact collagen and also failed to promote vascular tube formation. Together, these studies suggest that damage to the collagenous matrix is itself an important inducer of new vessel formation.

The process of new vessel formation (angiogenesis) involves localised breakdown of the vascular basement membrane, migration of endothelial cells into the surrounding stroma, endothelial cell proliferation and, eventually, organisation of the endothelial cells into a tubal structure with a central lumen (Singer and Clark, 1999; Cavallaro and Christofori, 2000; Stupack and Charesh, 2004). Soluble peptides such as vascular endothelial cell growth factor, hepatocyte growth factor and platelet-derived growth factor are thought to underlie the changes in endothelial cell physiology that ‘drive’ the angiogenic response (Lee *et al*, 2005; Rahman *et al*, 2005; Berthod *et al*, 2006). Altered expression of integrin receptors (Stupack and Charesh, 2004; Wang and Milner, 2006; Carnevale *et al*, 2007) and upregulation of surface proteolytic enzymes (Chun *et al*, 2004) accompany the response. Presumably, enzyme function is related to localised remodelling, but it is also possible that the enzymes play a more subtle role, for example, by releasing peptide growth factors from the matrix (Lee *et al*, 2005).

The angiogenic response is a prominent feature in several pathophysiological processes, including tumour invasion (Singer and Clark, 1999; Cavallaro and Christofori, 2000; Stupack and Charesh, 2004). During tumour invasion, new vessels form in the existing collagenous matrix at the primary tumour site and where invasion is occurring (Cavallaro and Christofori, 2000; Baronas-Lowell *et al*, 2004). Connective tissue damage is also a consistent feature of tumour invasion. We show in the present study, based on a series of *in vitro* observations, that damage to type I collagen, mediated largely by matrix metalloproteinase-1 (MMP-1; interstitial collagenase), provides an environment that stimulates the formation and in-growth of new blood vessels.

## MATERIALS AND METHODS

### Proteolytic enzymes and other reagents

Human MMP-1 (interstitial collagenase) was obtained from Calbiochem (San Diego, CA, USA). The enzyme was purified from human rheumatoid synovial fibroblasts as the naturally occurring proenzyme form. The MMP-1 preparation appeared as a doublet at 52 and 57 kDa in *β*-casein zymography, and it was reactive with a rabbit polyclonal anti-MMP-1 antibody (AB806; Chemicon International, Temecula, CA, USA) by western blotting. Activation of the proenzyme was accomplished by exposure of the latent enzyme to 1 *μ*g of crystalline trypsin for 5 min at 37°C followed by 10 *μ*g of soya bean trypsin inhibitor (SBTI). A mouse monoclonal antibody with neutralising activity for MMP-1 (Ab-5; IM66) was obtained from Oncogene Research Products (San Diego, CA, USA).

Matrix metalloproteinase-9 (92-kDa gelatinase B) was obtained as a recombinant protein (active form) produced in mammalian cells (Calbiochem). In gelatin zymography, active MMP-9 was seen as an 83-kDa moiety. Human recombinant tissue inhibitor of metalloproteinase-1 (TIMP-1) was also obtained from Calbiochem. Crystalline bovine pancreatic trypsin, hog pancreatic elastase and SBTI were obtained from Sigma Chemical Co. (St Louis, MO, USA).

### MMPs from human skin

Human basal cell carcinoma tissue was used as a source of human skin organ culture fluid. The use of discarded tumour tissue was approved by the University of Michigan Institutional Review Board and obtained through the Tissue Procurement Core at the University of Michigan Comprehensive Cancer Center. Fresh tumour specimens obtained at surgery were minced into small pieces and incubated for 3 days at 37°C in an atmosphere of 95% air and 5% CO_2_. Keratinocyte basal medium (KBM) (Lonza, Walkersville, MD, USA) supplemented with calcium chloride to a final concentration of 1.4 mM was used as culture medium. At the end of the incubation period, the culture fluid was harvested and clarified by low-speed centrifugation. Culture fluids from several tissues were ‘pooled’, aliquoted and stored at −80°C. Our previous studies have characterised the profile of MMPs in organ culture fluid from basal cell tumours (Varani *et al*, 2000; Monhian *et al*, 2005; Yucel *et al*, 2005). The culture fluids contain a large amount of active MMP-1 but virtually no MMP-8 (neutrophil collagenase) or MMP-13 (collagenase-3). Active forms of the two major gelatinolytic enzymes (e.g., MMP-2 (gelatinase A) and MMP-9) are also present in these conditioned media. Before use, each pooled preparation of culture fluid was assessed for total and active MMP-1 by *β*-casein zymography and for total and active MMP-2 and MMP-9 by gelatin zymography. In addition, collagen and gelatin fragmentation assays were used to assess (and standardise) overall collagenolytic and gelatinolytic activities in each preparation. The enzyme characterisation studies were performed exactly as described in our past reports (Varani *et al*, 2000; Brennan *et al*, 2003; Yucel *et al*, 2005).

### Preparation and degradation of polymerised collagen lattices

Three-dimensional lattices of reconstituted polymerised collagen were prepared as described previously (Varani *et al*, 2001). Rat tail collagen (3.7–4.7 mg ml^−1^ in 1 N HCl) (BD Biosciences, Bedford, MA, USA) was diluted to 2 mg ml^−1^ in culture medium consisting of serum-free, Ca^2+^-supplemented KBM. The collagen solution was made isotonic by addition of an appropriate amount of 10 × concentrated Hanks’ balanced salt solution and brought to pH 7.2. The collagen was added to wells of a 24-well plate (0.5 ml per well) and incubated for 2 h at 37°C, during which time a stiff lattice of polymerised collagen formed.

Degradation of the polymerised collagen was achieved by exposing the collagen lattice to human skin organ culture fluid or to purified MMP-1 for 18 h at 37°C. Intact collagen lattices exposed to buffer alone served as control. At the end of the incubation period, the supernatant fluids from control or treated collagen lattices were removed. Collagen fragmentation was assessed by measuring the peptides released into the supernatant fluid (indicated by sodium dodecylsulphate–polyacrylamide gel electrophoresis (SDS–PAGE) resolution and by staining with Coomassie brilliant blue). The polymerised collagen lattices (still intact) were then rinsed carefully with Ca^2+^-supplemented KBM. (Note: Although exposure of the collagen to high enzyme concentrations or exposure for an extended period of time to lower enzyme amounts would eventually result in complete solubilisation of the lattices, the enzyme amounts and incubation times used in these experiments produced no measurable change in the gross appearance of the collagen lattices as compared to collagen lattices exposed to buffer alone.)

### Endothelial cell behaviour on intact and damaged collagen

An immortalised line of rat endothelial cells was used in these studies. Dulbecco's modified Eagle's medium (DMEM) supplemented with non-essential amino acids and 10% fetal bovine serum (FBS) was used as culture medium (DMEM–FBS). Cells were maintained in monolayer culture at 37°C in an atmosphere of 5% CO_2_ and 95% air, and subcultured as required. The cells have been characterised in several past reports (reviewed by Varani and Ward, 1994). The cells form a cobblestone monolayer on a rigid plastic surface and continue to efficiently take up fluorescent-labelled low-density lipoprotein.

To assess endothelial cell capacity to organise into a network of tubes, the cells were harvested from monolayer culture and added to intact or partially degraded collagen lattices in DMEM–FBS. Cells were incubated at 37°C in an atmosphere of 95% air and 5% CO_2_ for 10 days (unless otherwise stated) with fresh culture medium added at 2-day intervals. At the end of the incubation period, the cultures were flooded with 10% buffered formalin. Endothelial cell tube formation was assessed microscopically, and the number of visible tubes counted per × 100 field. After counting, the cultures were photographed. In some cases, the cultures were fixed in 2% glutaraldehyde rather than formalin and used for scanning electron microscopy.

### Scanning electron microscopy

Intact and partially degraded collagen lattices were fixed by mixing with an equal volume of 4% Sorenson's buffered glutaraldehyde. Post-fixation in 1% osmium tetroxide buffered in *s*-collidine was followed by *en bloc* staining with uranyl acetate. Dehydration was performed in a graded series of ethanol, followed by critical point drying from absolute ethanol through liquid carbon dioxide. Specimens were then mounted on stubs and conductive coated with gold in a DC sputter coater. Following this, specimens were examined using an ISI Super IIIA scanning electron microscope. Scanning electron microscopy services were provided on a fee-for-service basis through the Morphology Core Laboratory in the Department of Cellular and Developmental Biology at the University of Michigan.

### Statistical analysis

Data from experiments with multiple groups were analysed using one-way analysis of variance (ANOVA), followed by the Bonferroni post-test for selected pairs (GraphPad Prism Version 4 for Windows; GraphPad Software, San Diego, CA, USA). For experiments in which there were only two groups, the Student's *t*-test was used to assess statistical significance of the differences. Data were considered significant at *P*<0.05.

## RESULTS

### Organisation of endothelial cells into vascular tubes on partially degraded collagen

**Figure 1:**
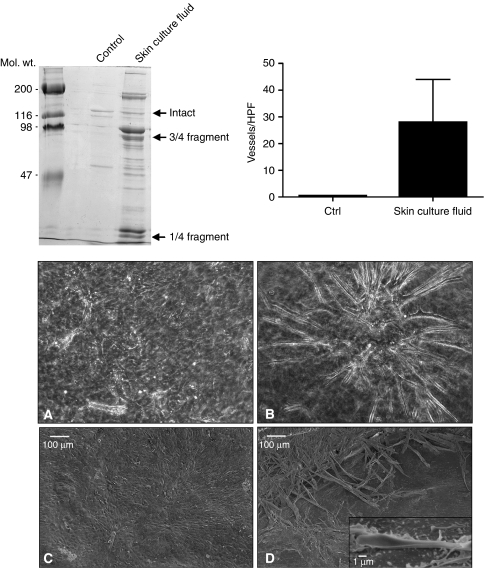
Organisation of endothelial cells into vascular tubes on partially degraded collagen. Upper left panel: SDS–PAGE analysis of the collagen fragmentation pattern obtained following exposure of intact collagen lattices to control buffer alone or to skin organ culture fluid. The collagen lattice exposed to buffer alone (control lane) shows a faint doublet corresponding to the *α*1 and *α*2 chains of intact type I collagen. The bands are faint because most of the intact collagen remains polymerised in the lattice. The collagen lattice exposed to organ culture fluid from UV-irradiated skin shows bands corresponding to the $3over 4$- and $1over 4$-sized fragments of type I collagen. Additional fragments are observed. Upper right panel: Number of vessels counted per high-power field on collagen lattices after 10 days of incubation. Values are means and standard deviations based on five separate experiments. Values were compared using Student's *t*-test. Differences between the two groups were significant at the *P*<0.01 level. Lower panels: Phase-contrast photomicrographs (**A** and **B**) and scanning electron micrographs (**C** and **D**) taken 10 days after plating of endothelial cells on intact and partially degraded collagen lattices. On the intact collagen, a monolayer of endothelial cells is seen (**A** and **C**). On the fragmented collagen, some of the endothelial cells are organised into a tubal network (**B** and **D**). The inset in panel D shows a tube with a clearly defined lumen.

In the first series of experiments, polymerised collagen lattices were exposed to organ culture fluid from either UV-irradiated skin or basal cell carcinoma tissue. After preliminary studies identified appropriate enzyme concentrations and digestion times, conditions were chosen (18-h digestion with 50 *μ*l of culture fluid per 1 mg of collagen in 0.5 ml) that did not cause the polymerised matrix to dissolve but resulted in partial conversion of the polymerised collagen into soluble fragments. Figure 1 (upper left) shows the typical fragmentation pattern seen after digestion. Consistent with past reports (Varani *et al*, 2000, 2001, 2002; Brennan *et al*, 2003; Monhian *et al*, 2005; Yucel *et al*, 2005), the $3over 4$- and $1over 4$-sized fragments that are the principal products of interstitial collagenase digestion of type I collagen were the predominant species identified. Not surprisingly, as significant amounts of active MMP-2 and MMP-9 (as well as other enzymes) are also present in skin organ culture fluid, fragments of other sizes were also detected. By quantitatively comparing the amount of fragments released into the culture fluid during partial digestion with a standard curve prepared after complete digestion of a comparable collagen matrix, it was determined that approximately 30% of the initial collagen was converted into fragments under the conditions of the experiment depicted in Figure 1.

Following digestion of the collagen and washing to remove the residual enzyme, endothelial cells (1.5 × 10^5^ per well) were added to the surface of intact and partially degraded collagen lattices. After incubation for 10 days, endothelial cell tube formation was assessed. Upper right panel of Figure 1 presents quantitative results from this experiment, and lower panels of Figure 1 show the appearance of the cells by phase-contrast and scanning electron microscopy. As can be seen from the bar graph (upper right) and the control photographs (lower panels, Figure 1A and C), there were essentially no vessel sprouts on the intact collagen after 10 days. Rather, it can be seen by both light and scanning electron microscopy that on intact collagen, the endothelial cells formed a dense monolayer of cells. In contrast, when endothelial cells were incubated on partially degraded collagen, a network of branched vessels developed. This can be seen quantitatively in the bar graph (upper right) and by phase-contrast and scanning electron microscopy (lower panels, Figure 1B and D). Most of the vessels appeared to run along or near the surface of the collagen matrix. At the scanning electron microscopic level, it could be seen that the tiny vessels were closed along much of the tube but open near the terminus – at the surface of the collagen (inset in Figure 1D).

**Figure 2:**
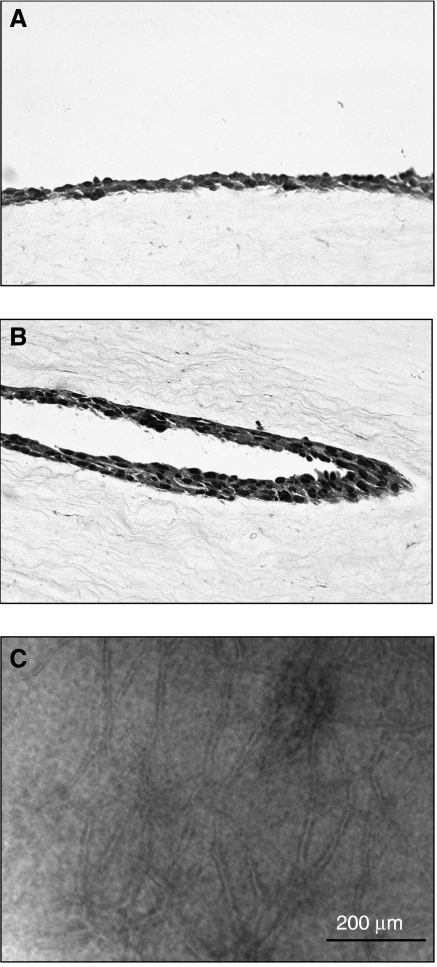
Evidence of vascular tube formation on partially degraded collagen lattices. (**A**) Monolayer of endothelial cells on the surface of an intact collagen lattice. (**B**) Vascular tube shown in longitudinal section within a partially degraded collagen lattice. Notice that the orientation of the tube has the same orientation as the surrounding collagen fibres. (**A**, **B**) Haematoxylin- and eosin-stained, 5-*μ*m-thick sections obtained after formalin fixation and paraffin imbedding are shown. (**C**) Phase-contrast photomicrograph of a partially degraded collagen lattice in which the plane of focus is 50–100 *μ*m below the surface of the lattice.

Standard histological approaches were used to visualise tube formation within the collagen lattice. Figure 2A and B presents sections through intact and partially digested collagen lattices. A dense monolayer of cells can be seen at the surface of the lattice in Figure 2A. There is no evidence of tube formation either at the surface or within the lattice itself. Figure 2B shows a section through a partially digested collagen lattice. A portion of an endothelial tube in longitudinal section can be seen imbedded in the collagen. The orientation of the tube is in the direction of the surrounding collagenous fibres. Figure 2C shows a phase-contrast image of a partially digested lattice. The plane of focus is below the surface of the lattice and the network of tubes (in focus) resides approximately 100 *μ*m below the surface. As can be seen from Figure 2C, a network of tubes of 800 *μ*m or more in length was not uncommon.

Additional experiments were carried out to assess the degree of collagen digestion that was optimal for vascular tube formation. For this, a standard curve was generated by complete solubilisation of a collagen lattice as described above. Using the standard curve for comparison, it was shown that when less than 10% of the collagen was digested, vessels did not form. Tube formation increased with increasing collagen digestion between 10 and 30%, but above 30% vessel formation was retarded. Rather, in the presence of excessive collagen fragmentation, the endothelial cells aggregated at the collagen surface (data not shown). When 50% or more of the collagen was digested, the polymerised lattice did not remain physically intact.

Cell number was also critical. When 5 × 10^4^ cells were added per culture (as opposed to the usual 1.5 × 10^5^), the formation of tubes was retarded, although the cells tended to align with one another rather than form a normal monolayer (data not shown).

Finally, time-course studies revealed that vascular sprouts could be seen within 3–4 days after plating on partially degraded collagen. The size and length of the endothelial cell tubes increased over the next several days. Beyond day 10, however, the number and size of the existing tubes increased very little, although the network of vessels that had formed remained stable for at least 2 additional weeks (longest period observed).

### Characterisation of enzyme(s) responsible for collagen degradation and formation of the vascular network

**Figure 3:**
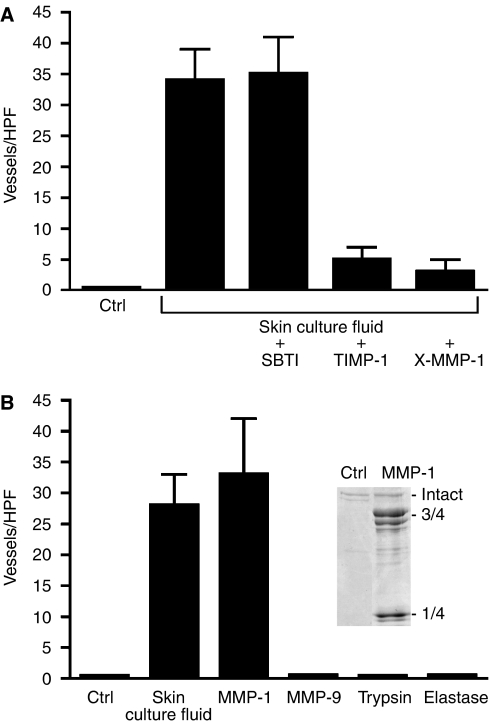
Characterisation of the enzyme(s) responsible for collagen degradation and vascular tube formation. (**A**) Collagen lattices were exposed to buffer alone (control) or to skin organ culture fluid that had been pretreated with either buffer alone, SBTI, TIMP-1 or a neutralising antibody to MMP-1. Endothelial tube formation was then assessed. Values are means and standard deviations based on three separate experiments. Values were analysed by ANOVA followed by paired group comparisons. Differences between the TIMP-1 and anti-MMP-1 groups and the positive control group (skin organ culture) were significant at the *P*<0.05 level. (**B**) Collagen lattices were exposed to buffer alone or to each of the enzymes indicated. Values are means and standard deviations based on three separate experiments with each enzyme. Values were analysed by ANOVA followed by paired group comparisons. Differences between the skin culture and MMP-1 groups and the control group were significant at the *P*<0.05 level.

A series of studies were carried out to characterise the enzyme(s) responsible for collagen degradation and induction of vessel formation. First, it was demonstrated that both collagen degradation and endothelial tube formation were unaffected when skin organ culture fluid was incubated with SBTI (5 *μ*g per 50 *μ*l of culture fluid) (Figure 3A). In contrast, incubation of the organ culture fluid with TIMP-1 (150 ng per 50 *μ*l of culture fluid) or pretreatment with a neutralising antibody to MMP-1 (50 *μ*g of antibody protein) completely suppressed collagen degradation and subsequent formation of endothelial tubes (Figure 3A).

Next, polymerised collagen lattices were exposed to MMP-1 (100 ng of activated enzyme) or to MMP-9, hog pancreatic elastase or bovine pancreatic trypsin (100 ng of each enzyme). Matrix metalloproteinase-1 degraded the polymerised collagen, producing the expected $3over 4$- and $1over 4$-sized cleavage products (Figure 3B, inset). When endothelial cells were incubated on MMP-1-degraded collagen, organisation into a branched network of tubes similar to that seen with skin organ culture fluid occurred (Figure 3B). In contrast, neither MMP-9 nor either of the serine proteinases produced measurable degradation of intact collagen (data not shown), and none of the enzymes stimulated vascular tube formation (Figure 3B).

### Failure of proteolytic fragments to modulate endothelial tube formation

**Figure 4:**
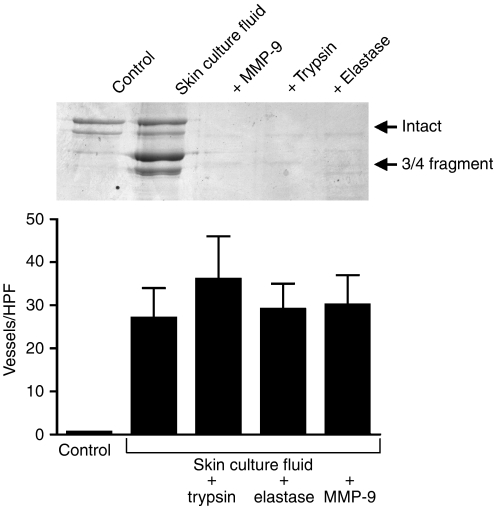
Clearance of skin culture fluid-generated fragments does not inhibit vascular tube formation. Collagen lattices were exposed to buffer alone or to organ culture fluid alone or in the presence of additional enzymes. Endothelial tube formation was then assessed in the normal manner. Values are means and standard deviations based on three separate experiments. Values were analysed by ANOVA followed by paired group comparisons. Differences between all of the treatment groups and the negative control group were statistically significant at the *P*<0.01 level. The inset shows that all three enzymes (MMP-9, trypsin or elastase) were able to further degrade and clear the fragments initially generated.

Experiments were carried out in which partial degradation of the collagen lattice was produced by exposure to human skin culture fluid in the normal manner. Additional collagen lattices were treated with the same culture fluid and concomitantly with MMP-9 (100 ng ml^−1^), trypsin (10 ng ml^−1^) or elastase (10 ng ml^−1^) to further degrade the fragments generated by the enzymes in the organ culture fluid. The inset of Figure 4 shows the pattern of fragments generated from the collagen by skin organ culture fluid alone and in the presence of exogenous MMP-9, trypsin or elastase. Further degradation and clearing of the fragments by MMP-9 and both serine proteinases are evident. Figure 4 shows tube formation on these substrates. Endothelial cell organisation into a network of tubes occurred equally well whether or not the skin culture fluid-generated fragments of collagen were further degraded and cleared.

**Figure 5:**
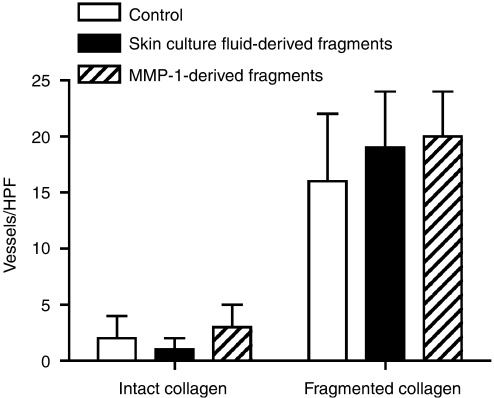
Failure of proteolytic fragments to modulate vascular tube formation. Collagen lattices were exposed to buffer alone (control) or to skin organ culture fluid. Cells were added to the intact or partially degraded lattices in the presence of buffer alone or in the presence of 300 *μ*g of collagen fragments from skin culture fluid-digested collagen or MMP-1-digested collagen. Endothelial tube formation was then assessed. Values are means and standard deviations based on three separate experiments.

In companion studies, experiments was carried out to determine if the proteolytic fragments generated from polymerised collagen would promote organisation of endothelial cells into vascular tubes on intact collagen, or, conversely, if the fragments would interfere with vascular tube formation on partially degraded collagen. Intact collagen was exposed to a sufficient level of human skin culture fluid (150 *μ*l) or MMP-1 (100 ng) to induce complete solubilisation of the collagen lattice. Next, additional collagen lattices were prepared, and some of these were partially degraded in the usual manner by exposure to skin organ culture fluid. Following this, endothelial cells were added to the intact and partially degraded collagen in the presence or absence of additional soluble fragments. For this experiment, 300 *μ*g of fragments (equivalent to the amount of fragments generated when 30% of the intact collagen matrix was digested) were used. As shown in Figure 5, soluble fragments generated from intact collagen neither stimulated vascular tube formation on intact collagen nor inhibited tube formation on partially degraded collagen.

### Failure of mechanically relaxed collagen lattices to support endothelial tube formation

A final set of experiments was carried out in which collagen lattices were mechanically relaxed by physically detaching the collagen matrix from the rim of the culture dish. For these studies, cells were added to intact collagen lattices and allowed to attach. Four hours later, individual collagen lattices were detached over 25, 50, 75 or 100% of the rim. As seen in Table 1, mechanical release of the collagen from contact with the edge of the plastic dish had minimal effect on tube formation.

## DISCUSSION

Studies in the past two decades have clearly established the capacity of cultured endothelial cells to form a branching network of vascular tubes *in vitro* under appropriate conditions. A three-dimensional matrix is critical; tube formation occurs in a polymerised three-dimensional lattice of collagen, but organisation of endothelial cells into capillary-like structures does not occur on a rigid plastic surface, even when the surface is coated with an appropriate matrix component (Stupack and Charesh, 2004; Wang and Milner, 2006; Carnevale *et al*, 2007). In spite of extensive efforts characterising the process of vascular tube formation *in vitro*, the relationship between connective tissue damage and tube formation has not been fully assessed. This is surprising because the biological processes in which vessel formation occurs are characterised by extensive connective tissue damage and remodelling. The data presented here show that damage to the collagenous matrix is a potent stimulus for endothelial cell organisation into a branching tubal structure.

Matrix metalloproteinase-1 appears to be the enzyme responsible for the majority of the collagen damage leading to endothelial tube formation. The fragmentation pattern produced from intact type I collagen by exposure to organ culture from either UV-treated skin or basal cell tumour tissue was characteristic of MMP-1 (i.e., with $3over 4$- and $1over 4$-sized fragments being the major degradation products). Both TIMP-1 and a neutralising antibody to MMP-1 effectively blocked collagen degradation, whereas SBTI had no effect. Additionally, fibroblast-derived MMP-1 duplicated the effects of the skin culture fluid, whereas three other enzymes did not. Finally, in previous studies, we showed that there was little MMP-8 (neutrophil collagenase) and MMP-13 (collagenase-3) in these culture fluids and that blocking the functional activity of these enzymes with specific antibodies had no significant effect on collagen degradation (Varani *et al*, 2002). Our finding that MMP-1 appears to be the critical enzyme leading to tube formation in no way conflicts with past studies showing a role for endothelial cell-surface MT1-MMP in angiogenesis (Chun *et al*, 2004). MT1-MMP appears to function primarily in local matrix remodelling and contact guidance during the actual invasion by endothelial cells.

How damage to the collagen matrix provokes endothelial cell organisation into a tubal network is not clear. Multiple factors may be involved. One factor may be a reduction in collagen density following enzyme exposure. Our working hypothesis is that endothelial cells are able to more easily penetrate the ‘less dense’, partially degraded connective tissue. Equally important, cell–cell interactions necessary for endothelial cell organisation into a lumen-containing structure may be facilitated in this environment. Although this was not the focus of the present study, a previous study demonstrated that damage to type IV collagen exposes cryptic sites that alter interactions with endothelial cells (Xu *et al*, 2001). On proteolytic enzyme-cleaved type IV collagen, there was reduced binding through *α*1*β*1 integrin and increased binding through *α*v*β*3 integrin. Concomitantly, endothelial cell adhesion and motility (properties required for angiogenesis) were altered. Finally, we think it is reasonable to suggest that a reduction in the rigidity of the surrounding matrix may contribute to endothelial cell organisation into tubal structures by reducing mechanical stress on the cells. However, as the data summarised in Table 1 clearly indicate, reducing mechanical stress by releasing the collagen lattice from the plastic dish is not sufficient, in and of itself, to promote tube formation. It may be that reduced matrix density and reduced rigidity in conjunction with altered signalling may all be required for an angiogenic response. Additional factors not considered here may also contribute to the process of vessel formation (Davis and Senger, 2005). Our data do not indicate otherwise. Rather, our findings simply suggest that the microenvironment created by connective tissue degradation is itself a stimulus for new vessel formation to occur.

That damaged connective tissue is such a powerful stimulus to altered endothelial cell function should not be entirely unexpected. We have shown in a recent series of studies that matrix damage also has a profound effect on the functioning of imbedded fibroblasts (Varani *et al*, 2001, 2002, 2004, 2006; Fligiel *et al*, 2003). When fibroblasts were maintained on intact collagen lattices, they showed a flattened, well-spread appearance (similar to what was seen in monolayer cultures on plastic). Fibroblast attachment to collagen bundles occurred at numerous points (indicated by the presence of multiple focal adhesions) and the cells showed a high degree of mechanical stress (indicated by actin stress fibre formation). When the collagen was partially degraded using a protocol similar to that used here, fibroblasts attached to the damaged matrix as readily as to the intact substrate. However, they did not flatten out. Rather, these cells remained spherical; there were few focal adhesions and few actin stress fibres. Fibroblasts maintained on intact collagen synthesised a high level of type I procollagen and a low level of MMP-1. On the partially degraded substrate, procollagen production decreased dramatically (60–80%) and MMP levels increased. Based on these findings, we speculated that the fragmented collagen had lost its ability to provide structural support for the imbedded cells and that this resulted in a loss of mechanical tension on the cells. Consistent with previous *in vitro* studies (Clark *et al*, 1995; Chiquet, 1999), our data indicated that a synthetic phenotype depended in some manner of the mechanical tension resulting from interaction between the fibroblasts and the surrounding three-dimensional support. Of interest, the phenotype of interstitial fibroblasts on intact collagen mimicked the phenotype of fibroblasts in healthy young skin, whereas the phenotype on damaged collagen was similar to that seen in both photodamage (Varani *et al*, 2004) and chronological ageing (Varani *et al*, 2006).

Although damaged collagen influences the behaviour of both endothelial cells and fibroblasts, important differences distinguish the two cell types in their response to the fragmented connective tissue. Contraction of the damaged matrix and decreased collagen synthesis were the primary responses seen with dermal fibroblasts. With fibroblasts, the more extensive the collagen damage, the more contraction of the collagen occurred, and the greater was the decrease in collagen production. With endothelial cells, in contrast, it appears that the amount of collagen damage required to support vascular tube formation needs to be within precise limits. When there was too little matrix damage, cells formed a monolayer similar to what is typically seen on plastic. When damage to the matrix was too extensive, endothelial cells grew as a packed colony of cells. The cells failed to migrate across the surface of the damaged matrix, and no tubal structures were formed.

Another difference between fibroblasts and endothelial cells in their interaction with damaged collagen may be inferred from studies in which the fragments of collagen generated initially were further degraded and cleared. Our earlier fibroblast studies indicated that when MMP-1-generated fragments of type I collagen were further degraded and cleared by exposure to MMP-9, mechanical tension on the cells remained high as the cells had only intact collagen with which to interact (Varani *et al*, 2002). Collagen synthesis also remained high. In contrast, the present studies showed that further degradation of the collagenase-generated fragments neither induced nor hindered vascular tube formation. Likewise, the addition of soluble collagenase-derived fragments to endothelial cells on intact or partially degraded collagen also had no effect on tube formation. As already noted, our interpretation of these results is that vascular tube formation is not simply an effect of reduced mechanical stress *per se*, but more likely, depends on multiple signals emanating from the damaged matrix.

Connective tissue damage and angiogenesis are consistent features of the tumour invasion process. In the skin, both basal epithelial tumours and squamous epithelial tumours produce a large amount of tissue damage. Collagen destruction is visible ultrastructurally and biochemically in these tumours (Monhian *et al*, 2005; Yucel *et al*, 2005), and new vessels are prominent in the partially degraded connective tissue. In past studies from our laboratory, we showed that the epithelial tumour cells themselves, but especially the surrounding fibroblasts, produce large amounts of MMP-1. We suggest, based on the data presented here, that MMP-1-mediated connective tissue damage in the tumour matrix itself provides the stimulus for new vessel formation.

## Figures and Tables

**Table 1:** Failure of mechanically relaxed collagen lattices to support vascular tube formation

**Percentage of lattice released**	**Vessels/HPF**
0	4±2
25	1±2
50	3±1
75	2±3
100	2±1

HPF=high-power field.

The collagen lattices were prepared in the normal manner and cells added. After the cells were firmly attached, a 26-gauge needle was used to carefully detach the collagen from the edge of the plastic culture dish over the desired amount. Tube formation was then assessed at day 10 in the normal manner. Values are means and standard deviations based on three separate experiments.

## References

[bib1] Baronas-Lowell D, Lauer-Fields JL, Fields GB (2004) Induction of endothelial cell activation by a triple helical alpha2beta1 integrin ligand derived from type I collagen alpha1496-507. J Biol Chem 279: 952–962 14581484 10.1074/jbc.M305989200

[bib2] Berthod F, Germain L, Tremblay N, Auger FA (2006) Extracellular matrix deposition by fibroblasts is necessary to promote capillary like tube formation *in vitro*. J Cell Physiol 207: 491–498 16453301 10.1002/jcp.20584

[bib3] Brennan M, Bhatti H, Nerusu KC, Bhagavathula N, Kang S, Fisher GJ, Voorhees JJ, Varani J (2003) Matrix metalloproteinase-1 is the major collagenolytic enzyme responsible for collagen damage in UV-irradiated human skin. Photochem Photobiol 78: 43–48 12929747 10.1562/0031-8655(2003)078<0043:mmitmc>2.0.co;2

[bib4] Carnevale E, Fogel E, Alpin AC, Gelati M, Howson KM, Zhu WH, Nicosia RF (2007) Regulation of postangiogenic neovessel survival by beta 1 and beta 3 integrins in collagen and fibrin matrices. J Vasc Res 44: 40–50 17167269 10.1159/000097976

[bib5] Cavallaro U, Christofori G (2000) Molecular mechanisms of tumor angiogenesis and tumor progression. J Neurooncol 50: 63–70 11245282 10.1023/a:1006414621286

[bib6] Chiquet M (1999) Regulation of extracellular gene expression by mechanical stress. Matrix Biol 18: 417–426 10601729 10.1016/s0945-053x(99)00039-6

[bib7] Chun TH, Sabeth F, Ota I, Murphy H, McDonagh KT, Holmbeck K, Dirkedal-Hansen H, Allen ED, Weiss SJ (2004) MT1-MMP-dependent neovessel formation within the confines of a three-dimensional extracellular matrix. J Cell Biol 167: 757–767 15545316 10.1083/jcb.200405001 PMC2172577

[bib8] Clark RAF, Nielsen LD, Welch MP, McPherson JM (1995) Collagen matrices attenuate the collagen-synthetic response of cultured fibroblasts to TGF-*β*. J Cell Sci 108: 1251–1261 7622608 10.1242/jcs.108.3.1251

[bib9] Davis GE, Senger DR (2005) Endothelial extracellular matrix: biosynthesis, remodeling and functions during vascular morphogenesis and neovessel stabilization. Circ Res 97: 1093–1107 16306453 10.1161/01.RES.0000191547.64391.e3

[bib10] Fligiel SEG, Varani J, Datta SH, Kang S, Fisher GJ, Voorhees JJ (2003) Collagen degradation in aged/photoaged skin *in vivo* and after exposure to MMP-1 *in vitro*. J Invest Dermatol 120: 842–848 12713591 10.1046/j.1523-1747.2003.12148.x

[bib11] Lee S, Jilani SM, Nikolova GV, Carpizo D, Irulela-Arispe ML (2005) Processing of VEGF-A by matrix metalloproteinases regulates bioavailability and vascular patterning in tumors. J Cell Biol 169: 681–691 15911882 10.1083/jcb.200409115 PMC2171712

[bib12] Monhian N, Jewett BS, Baker SR, Varani J (2005) Matrix metalloproteinase expression in normal skin associated with basal cell carcinoma and in distal skin from the same patients. Arch Facial Plast Surg 74: 238–243 10.1001/archfaci.7.4.238 16027344

[bib13] Rahman S, Patel Y, Murray J, Patel KV, Sumathipala R, Sobel M, Wijelath ES (2005) Novel hepatocyte growth factor (HGF) binding domains on fibronectin and vitronectin coordinate a distinct and amplified Met-integrin induced signalling pathway in endothelial cells. BMC Cell Biol 6: 8–17 15717924 10.1186/1471-2121-6-8 PMC553973

[bib14] Singer AJ, Clark RAF (1999) Cutaneous wound healing. N Engl J Med 341: 738–746 10471461 10.1056/NEJM199909023411006

[bib15] Stupack DG, Charesh DA (2004) Integrins and angiogenesis. Curr Top Dev Biol 64: 207–238 15563949 10.1016/S0070-2153(04)64009-9

[bib16] Varani J, Dame MK, Rittie L, Fligiel SEG, Kang S, Fisher GJ, Voorhees JJ (2006) Decreased collagen synthesis in chronologically aged skin: roles of age-dependent alterations in cellular aging and alterations in mechanical tension. Am J Pathol 168: 1861–1868 16723701 10.2353/ajpath.2006.051302 PMC1606623

[bib17] Varani J, Perone P, Fligiel SEG, Fisher GJ, Voorhees JJ (2002) Inhibition of type I procollagen production in photodamage: correlation between presence of high molecular weight collagen fragments and reduced procollagen synthesis. J Invest Dermatol 119: 122–129 12164934 10.1046/j.1523-1747.2002.01810.x

[bib18] Varani J, Schuger L, Dame MK, Leonard C, Fligiel SEG, Kang S, Fisher GJ, Voorhees JJ (2004) Reduced fibroblast interaction with intact collagen as a mechanism for depressed collagen synthesis in photodamaged skin. J Invest Dermatol 122: 1471–1479 15175039 10.1111/j.0022-202X.2004.22614.x

[bib19] Varani J, Spearman D, Perone P, Fligiel SEG, Datta SC, Wang ZQ, Shao Y, Kang S, Fisher GJ, Voorhees JJ (2001) Inhibition of type I procollagen synthesis by damaged collagen in photoaged skin and by collagenase-degraded collagen *in vitro*. Am J Pathol 158: 931–942 11238041 10.1016/S0002-9440(10)64040-0 PMC1850364

[bib20] Varani J, Ward PA (1994) Physiological basis of endothelial cell injury in acute inflammation. Shock 2: 311–319 7743355 10.1097/00024382-199411000-00001

[bib21] Varani J, Yiqing C, Datta SC, Zeigler ME (2000) Matrix metalloproteinase production by basal cell carcinoma skin and normal human skin in organ culture. Br J Cancer 82: 657–665 10682680 10.1054/bjoc.1999.0978 PMC2363319

[bib22] Wang J, Milner R (2006) Fibronectin promotes brain capillary endothelial cell survival and proliferation through alpha5beta1 and alphavbeta3 integrins via MAP kinase signalling. J Neurochem 96: 148–159 16269008 10.1111/j.1471-4159.2005.03521.x

[bib23] Xu J, Rodriguez D, Petitclerc E, Kim JJ, Hangai M, Yuen SM, Davis GE, Brooks PC (2001) Proteolytic exposure of a cryptic site within collagen type IV is required for angiogenesis and tumor growth *in vivo*. J Cell Biol 154: 1069–1079 11535623 10.1083/jcb.200103111 PMC2196184

[bib24] Yucel T, Mutnal A, Fay K, Fligiel SEG, Wang T, Johnson T, Baker SR, Varani J (2005) Matrix metalloproteinase expression in basal cell carcinoma: relationship between enzyme profile and collagen fragmentation pattern. Exp Mol Pathol 79: 151–160 16004981 10.1016/j.yexmp.2005.05.003

